# Development and assessment of a hand assist device: GRIPIT

**DOI:** 10.1186/s12984-017-0223-4

**Published:** 2017-02-21

**Authors:** Byungchul Kim, Hyunki In, Dae-Young Lee, Kyu-Jin Cho

**Affiliations:** 1 School of Mechanical and Aerospace Engineering/SNU-IAMD, Seoul National University, Seoul, 08826 Republic of Korea; 2 Korea Institute of Industrial Technology, Kimje, 55324 Republic of Korea

**Keywords:** Spinal cord injury, Assist device, Writing aids, Assessment, Exoskeleton

## Abstract

**Background:**

Although various hand assist devices have been commercialized for people with paralysis, they are somewhat limited in terms of tool fixation and device attachment method. Hand exoskeleton robots allow users to grasp a wider range of tools but are heavy, complicated, and bulky owing to the presence of numerous actuators and controllers. The GRIPIT hand assist device overcomes the limitations of both conventional devices and exoskeleton robots by providing improved tool fixation and device attachment in a lightweight and compact device. GRIPIT has been designed to assist tripod grasp for people with spinal cord injury because this grasp posture is frequently used in school and offices for such activities as writing and grasping small objects.

**Methods:**

The main development objective of GRIPIT is to assist users to grasp tools with their own hand using a lightweight, compact assistive device that is manually operated via a single wire. GRIPIT consists of only a glove, a wire, and a small structure that maintains tendon tension to permit a stable grasp. The tendon routing points are designed to apply force to the thumb, index finger, and middle finger to form a tripod grasp. A tension-maintenance structure sustains the grasp posture with appropriate tension. Following device development, four people with spinal cord injury were recruited to verify the writing performance of GRIPIT compared to the performance of a conventional penholder and handwriting. Writing was chosen as the assessment task because it requires a tripod grasp, which is one of the main performance objectives of GRIPIT.

**Results:**

New assessment, which includes six different writing tasks, was devised to measure writing ability from various viewpoints including both qualitative and quantitative methods, while most conventional assessments include only qualitative methods or simple time measuring assessments. Appearance, portability, difficulty of wearing, difficulty of grasping the subject, writing sensation, fatigability, and legibility were measured to assess qualitative performance while writing various words and sentences. Results showed that GRIPIT is relatively complicated to wear and use compared to a conventional assist device but has advantages for writing sensation, fatigability, and legibility because it affords sufficient grasp force during writing. Two quantitative performance factors were assessed, accuracy of writing and solidity of writing. To assess accuracy of writing, we asked subjects to draw various figures under given conditions. To assess solidity of writing, pen tip force and the angle variation of the pen were measured. Quantitative evaluation results showed that GRIPIT helps users to write accurately without pen shakes even high force is applied on the pen.

**Conclusions:**

Qualitative and quantitative results were better when subjects used GRIPIT than when they used the conventional penholder, mainly because GRIPIT allowed them to exert a higher grasp force. Grasp force is important because disabled people cannot control their fingers and thus need to move their entire arm to write, while non-disabled people only need to move their fingers to write. The tension-maintenance structure developed for GRIPIT provides appropriate grasp force and moment balance on the user’s hand, but the other writing method only fixes the pen using friction force or requires the user’s arm to generate a grasp force.

**Electronic supplementary material:**

The online version of this article (doi:10.1186/s12984-017-0223-4) contains supplementary material, which is available to authorized users.

## Background

The hand is one of the most essential body parts for independent living because so many tasks of daily life, such as writing, eating, and grasping, require a functional hand. People who suffer from permanent paralysis of the hand owing to cerebral palsy, spinal cord injury (SCI), stroke, and other neurological disorders require assistive or rehabilitation devices in order to regain independence and return to work [1, 2].

**Fig. 1:**
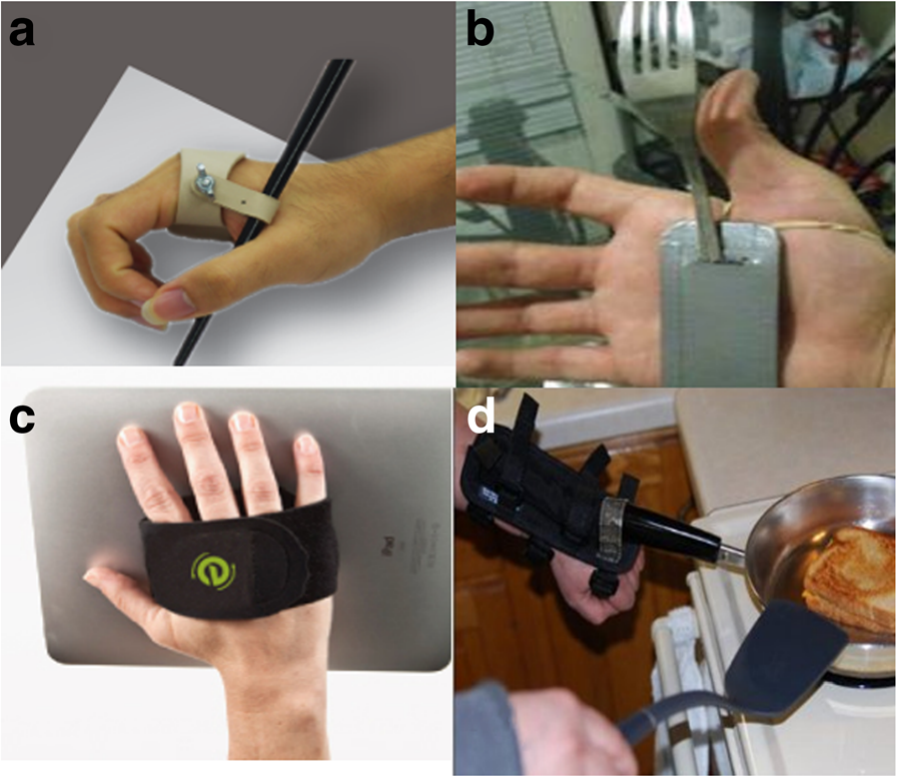
Various types of hand assist devices for people with hand paralysis. **a** Writing aid. **b** Eating aid. **c** Grasping aid. **d** Cooking aid

A selection of commercialized hand assist devices is shown in Fig. 1. These devices are attached to the user’s arm or hand with Velcro^®^ or elastic bands, and hand tools such as pens, forks, and paintbrushes are clamped into a hole in the devices. One drawback of these devices is that they can only grasp one type of tool because the receiving hole is a constant size. Users also must sometimes sustain an awkward posture to use a tool because it is mounted into the device in an unfamiliar position. Additionally, the Velcro or elastic band used to fix the device can apply high pressure to the skin if the strapping is too tight, and tools can be too shaky to use if the strapping becomes too loose. These problems reduce the usability of these devices and require users to put in a certain of amount of training time to become familiar with their use.

Hand exoskeletons, or wearable robots for the hand, have been developed to change the hand’s posture to facilitate tool use, instead of just strapping a tool to the hand. These exoskeletons do not have the disadvantages of conventional hand assist devices because they assist users to grasp tools by helping them to put their hand into a grasp posture. Hand exoskeletons can be classified in two ways, as robots employing rigid materials and robots employing flexible materials. Rigid exoskeletons use hard transmission such as linkages or gears and have the advantage of precise control due to the ease of implementing accurate model. However, rigid exoskeletons require joint alignment between the finger and the robot, making the wearing part bulky and heavy. For this reason, hard exoskeletons are mostly used for rehabilitation purposes [3–6]. Soft exoskeletons, on the other hand, have the advantage of simplifying the wearing part because they are easily locate actuator parts remotely that are separated from the worn portion. Most soft exoskeletons are used for assistance or home rehabilitation purposes because of said advantages [7–10].

In this paper we propose a soft wearable assistive device, GRIPIT, which enables people with spinal cord injuries, SCI, to attain one type of grasp via manual operation. By giving up assistance for various postures and tasks, GRIPIT has been able to reduce its complexity, weight, and volume compared to existing exoskeleton designs. GRIPIT was designed to operate manually, minimizing weight and volume by excluding controllers and actuators. Using a single wire to manually actuate the device reduces the difficulty of manual operation. GRIPIT, provides sufficient grasp stability compared to conventional assist devices because it helps the user grasp objects by applying forces through wire actuation. Developed using previously listed design goals, GRIPIT has a total weight of approximately 40 g and consists of a glove and a small circular structure with 30 mm diameter and 20 mm height [11].

We selected a tripod grasp as the posture that GRIPIT will assist because this grasp is frequently used in school and offices for such activities as writing and grasping small objects [12]. We focused on building an assist device for use in a school or office environment because the second most common age range for SCI is 15 to 29 years old, a time of life when many people are in school or building a career. Hand paralysis for people in this age range means an unavoidable change of occupation or serious problems with finishing their education [13, 14].

Four people with SCI were recruited to measure writing performance using GRIPIT, a conventional assist penholder (shown in Fig. 1a), and their own hand without any devices, because assisting writing is one of the major objectives of GRIPIT. The conventional penholder used in this experiment is the most frequently used device for this task. The device fixes the pen with a hole and is attached to the body with Velcro. We assessed the qualitative performance factors of appearance, portability, difficulty of use, writing sensation, fatigability, and legibility and the quantitative performance factors of writing accuracy and solidity of grasp.

## Methods

### Design approach: A glove assistance device using an underactuated tendon-driven system

The main development objective of GRIPIT is to assist users to make a stable tripod grasp posture with a mechanism that is compact, lightweight, and simple. Tripod grasp provides sufficient force with three fingers and moment balance with force applied by radial side of hand while other writing aids only provide friction force to fix the pen. The device uses an underactuated tendon-driven mechanism as its transmission method and a glove as the main frame of the device.

Tripod grasp is a precision grasp for compact objects that uses three fingers, according to the Cutkosky grasp taxonomy [15, 16]. The three fingers should be gathered into a single point, and the forces applied to an object should be well distributed for a stable grasp. The grasp can fail when the three fingers fail to gather or when one finger applies higher contact force to the object than the others. [17, 18].

The device uses a tendon-driven mechanism to form the fingers into a tripod grasp because of its compactness and light weight among various transmission mechanisms [19]. The underactuation mechanism, which is widely used in robotic hands and wearable hand robots to increase adaptability, is applied to the tendon-driven mechanism to gather the fingers into a single point and to distribute contact forces adaptably without complicated controlling of numerous DOF [9, 20, 21]. The tendon routing points were designed to create the proper posture with simple actuation, and a tension-maintenance structure keeps the grasp stable.

We chose to use a glove as the main frame of the device on which to fix the positions of the tendon routing points for the following reasons. The main frame should be closely attached to the user’s hand to increase the solidity of grasp; a glove readily permits this close attachment. In addition, the frame should not be loose or slip from the users hand for high solidity; these problems do not occur when the glove fits the user’s hand. SCI patients can easily fit their hand in glove due to hand size. Since muscle loss is a symptom of SCI, users hands have less girth. Therefore, finger length is the only constraint that requires consideration when choosing the glove. When the frame is rigid, the joints of the main frame must be aligned with the hand’s joints to apply force effectively. Ensuring coincidence of the center of rotation with the finger is a difficult problem because the flexion and extension motions of human joints involve combinations of rotation and transition motions. The center of rotation of a human joint continuously changes during movement [22]. However, these problems do not occur for the glove-based device because a glove is a soft structure without joint or link.

### Tendon routing design

**Fig. 2:**
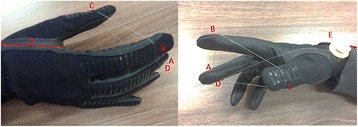
Prototype of GRIPIT showing the *single wire* and the location of the Teflon tubes. Wire is fixed at a point **a** and passes through **b**, **c** and **d**. *Dotted line* shows the wire path from the hand to the wrist

The tendon routing points are implemented with four Teflon™ tubes as follows: Two Teflon tubes are attached to the middle finger (a and d in Fig. 2), and a single Teflon tube is attached to the thumb (c in Fig. 2) and to the index finger (b in Fig. 2). A single wire is fixed at Teflon tube A and passes through B, C, and D. After passing point D at the middle finger, the wire enters the glove and leads to point E (dotted line in Fig. 2). The tendon entering D, into the glove, passes under the center of rotation of the middle finger joint, generating a flexion moment on the finger. GRIPIT applies force to the fingers to gather them into a single point when the user pulls the wire in the E direction, as shown in Fig. 2.

A tripod grasp will fail if one of the three fingers applies force to the object first or the force equilibrium breaks. However, GRIPIT cannot experience these problems because it uses a differential mechanism with underactuation: The other fingers start to move when one of the three fingers touches an object, and force equilibrium is always sustained because GRIPIT uses only a single wire. The three fingers always gather into a single point because the tendon pass has a closed shape.

GRIPIT uses a differential mechanism with an underactuated tendon-driven system to increase the adaptability of its grasp. GRIPIT assists users to gather three fingers to a single point without requiring precise force or position control.

### Tension-maintenance winder design

A tension-maintenance system is required for GRIPIT because the gripping posture loosens when the wire tension drops. We developed a tension-maintenance winder to sustain a constant wire tension, which maintains a stable grasp posture. The minimum tension required to form the writing posture was calculated for development of the tension-maintenance winder [22, 23]. The relation between tension and fingertip forces and between fingertip forces and pen tip force must be considered to calculate the proper tension. The required constant wire tension is determined from pen tip force measurement and the above relationships.

**Fig. 3:**
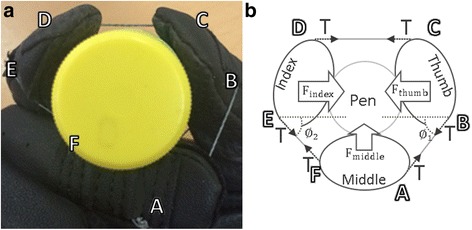
Schematic drawing of the tension calculation. **a** Labelled *tendon routing points*. **b** Schematic drawing of the free body diagram. T, wire tension; ∅1, ∅2, angles from the configuration of the wire path

Figure 3 shows a simplified schematic drawing of the relationship between wire tension and fingertip forces. Two assumptions are made to simplify the model. First, we ignored the friction force between the glove and the wire. It is because the Teflon tube, which has an extremely small friction coefficient, is imbedded along the wire path of glove. Second, the impedance of the human finger joint is neglected because it is significantly smaller than the tension applied by the wire [24]. Since an SCI patient cannot apply force to their finger, fingertip forces can only be determined by the tension of the wire and the posture of the hand using the above two assumptions. Figure 3a labels the tendon routing points. The wire is tied to point A on the middle finger and passes from point A to point F. Figure 3b is a schematic drawing of the free body diagram analysis. Eqs 1 to 3 deal with the relationships between the fingertip forces applied to the pen and the wire tension and were derived from the schematic drawing in Fig. 3b. T in the equation is the wire tension while grasping objects, ∅_1_ is an angle between wire $\overline{\mathrm{AB}}$ and $\overline{\mathrm{CD}}$, and ∅_2_ is an angle between $\overline{\mathrm{CD}}$ and $\overline{\mathrm{EF}}$.

1 $$ {\mathrm{F}}_{\mathrm{thumb}}=\mathrm{T}\left(1+ \cos {\varnothing}_1\right) $$

2 $$ {\mathrm{F}}_{\mathrm{index}}=\mathrm{T}\left(1+ \cos {\varnothing}_2\right) $$

3 $$ {\mathrm{F}}_{\mathrm{middle}}=\mathrm{T}\left( \sin {\varnothing}_1+ \sin {\varnothing}_2\right) $$

**Fig. 4:**
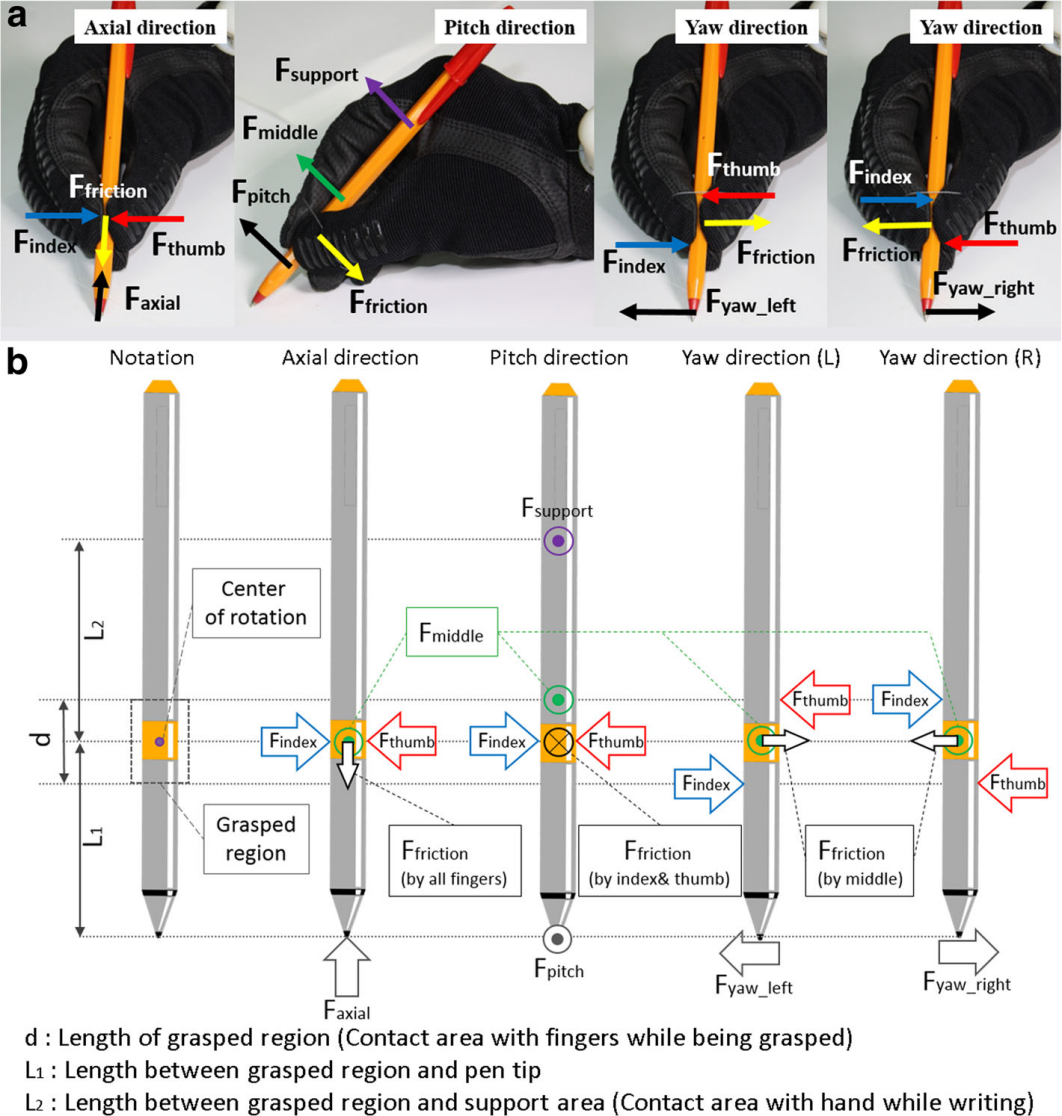
Derivation of the minimum fingertip force condition. **a** Force resolution in *axial*, *pitch,* and *yaw* directions. **b** Free body diagrams for each direction and the notations used in the figure

We analyzed the relations between pen tip force, the reaction force that the paper applies to the pen during writing, and the wire tension for the stable grasp condition. The pen tip force was divided into axial, pitch, and yaw directions for analysis, as shown in Fig. 4a. Minimum fingertip force conditions for each direction are solved in Eqs 4 to 7 using the force and momentum equilibrium of the free body diagrams in Fig. 4b. In Fig. 4b μ and d respectively are the friction coefficient and the length of a grasped region, which is a contacted area between the finger and the glove. L_1_ in Fig. 4b indicates the length between the grasped region and the pen tip. L_2_ in Fig. 4b is the length between the grasped region and the support point, which is the point of contact between the pen and the hand. Equation 4 is driven by the force equilibrium in the axial direction of the pen, and Eqs 5 to 7 are driven by the momentum equilibrium in each direction.

4 $$ \upmu \left({\mathrm{F}}_{\mathrm{index}}+{\mathrm{F}}_{\mathrm{thumb}}+{\mathrm{F}}_{\mathrm{middle}}\right)\ge {\mathrm{F}}_{\mathrm{axial}} $$

5 $$ {\mathrm{L}}_2\upmu \left({\mathrm{F}}_{\mathrm{index}}+{\mathrm{F}}_{\mathrm{thumb}}\right)-\left({\mathrm{L}}_2-0.5\mathrm{d}\right){\mathrm{F}}_{\mathrm{middle}}\ge \left({\mathrm{L}}_1+{\mathrm{L}}_2\right){\mathrm{F}}_{\mathrm{pitch}} $$

6 $$ 0.5\mathrm{d}\left({\mathrm{F}}_{\mathrm{index}}+{\mathrm{F}}_{\mathrm{thumb}}\right)\ge {\mathrm{L}}_1\ {\mathrm{F}}_{{\mathrm{yaw}}_{\mathrm{left}}} $$

7 $$ 0.5\mathrm{d}\left({\mathrm{F}}_{\mathrm{index}}+{\mathrm{F}}_{\mathrm{thumb}}\right)\ge {\mathrm{L}}_1\ {\mathrm{F}}_{{\mathrm{yaw}}_{\mathrm{right}}} $$

The minimum tension conditions for the axial, pitch, and yaw directions were calculated with Eqs 8 to 10 in Table 1, substituting the fingertip force shown in Eqs 1 to 3 for the fingertip force conditions for a stable grasp shown in Eqs 4 to 7. L_1_, L_2_, ∅_1_, ∅_2_ and d in Eqs 1 to 7 were measured to calculate the required tensions. The literature described range of the frictional coefficient between two nonmetal materials (0.4 < μ <0.9), and the maximum pen tip force (F_axial_ = 1.300, F_pitch_ = 1.390, F_yaw_ = 0.304) were also applied to obtain the tension conditions [25–28]. According to the results in Table 1, the minimum tension required to keep the grasp stable in all directions is 7.39 N.

**Table 1:** Calculation of the minimum tension condition and the required tension using the maximum pen tip force from the literature

	Minimum tension condition	Minimum tension for each condition (*N*)
Axial	${\mathrm{T}}_{\mathrm{axial}}\ge \frac{{\mathrm{F}}_{\mathrm{axial}}}{\upmu \left(2+\mathrm{Cos}{\varnothing}_1+\mathrm{Cos}{\varnothing}_2+\mathrm{Sin}{\varnothing}_1+\mathrm{Sin}{\varnothing}_2\right)}\kern0.6em (8)$	0.302–0.679
Pitch	${\mathrm{T}}_{\mathrm{pitch}}\ge \frac{\left({\mathrm{L}}_1+{\mathrm{L}}_2\right){\mathrm{F}}_{\mathrm{pitch}}}{{\upmu \mathrm{L}}_2\left(2+\mathrm{Cos}{\varnothing}_1+\mathrm{Cos}{\varnothing}_2\right)-\left(\mathrm{Sin}{\varnothing}_1+\mathrm{Sin}{\varnothing}_2\right)\left({\mathrm{L}}_2-0.5\mathrm{d}\right)}\kern0.85em (9)$	3.285–7.390
Yaw (left)	${\mathrm{T}}_{\mathrm{yaw}\_\mathrm{left}}\ge \frac{2{\mathrm{L}}_1\ {\mathrm{F}}_{{\mathrm{yaw}}_{\mathrm{left}}}}{\mathrm{d}\left(2+\mathrm{Cos}{\varnothing}_1+\mathrm{Cos}{\varnothing}_2\right)}\kern0.85em (10)$	3.7994

**Fig. 5:**
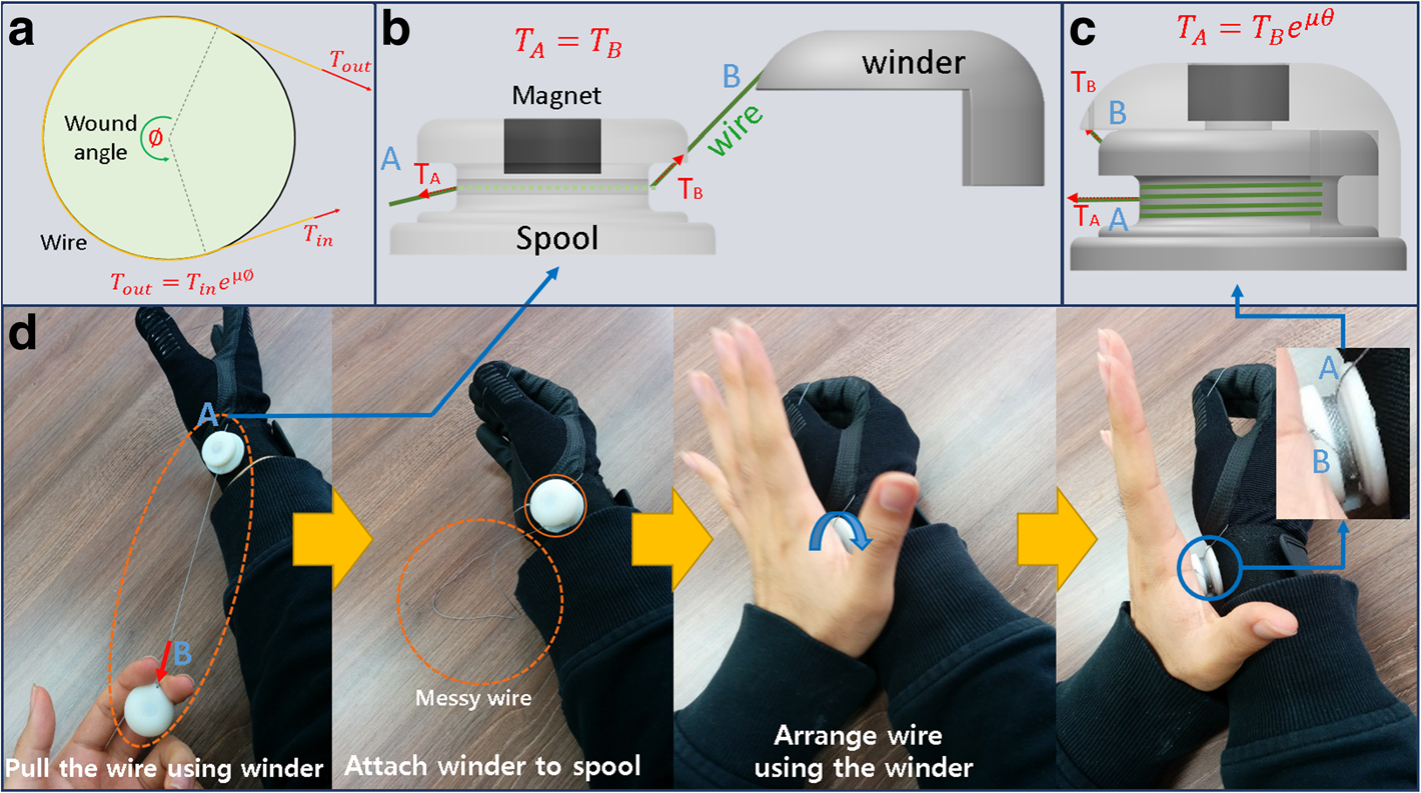
**a** Schematic drawing of the capstan equation, which is the main principle governing the tension-maintenance winder. **b** Schematic drawing of the process of *pulling* the wire. **c** Schematic drawing of the winder after the wire has been *wound up*. **d** Photographs illustrating use of the *winder*

We developed a tension-maintenance system using the capstan equation (Fig. 5a), which sustains a stable grasp posture after the required tension is attained. Figure 5 shows the winder, which is composed of one spool and one winder that holds the grasp force when the wire is wound around the pulley. The wire passes through a hole in the spool. One end of the wire is fixed at the winder (point B in Fig. 5), and the other end is fixed at the glove (point A in Fig. 5b). The process for using the tension-maintenance winder is shown in Fig. 5d. The wire passes through the spool when the user pulls the winder. After the fingers have been gathered into a grasp, the user can easily attach the winder to the spool by connecting the magnets on the spool and the winder. Messy wire is easily re-arranged by rotating the winder, and this process also generates the tension required for a stable grasp. Target users who have injuries to the C5 or C6 sections of their spine can use this tension-maintenance winder independently because they can use their wrist and arm.

Tension at point A (T_A_) can easily exceed 7.39 N, the minimum tension for a stable grasp, because the tension at point A exponentially increases when the wire is wound around the spool using the winder, according to the capstan equation shown in Fig. 5a (μ is the frictional coefficient between the pulley and the wire, and ∅ is the wound angle).

**Fig. 6:**
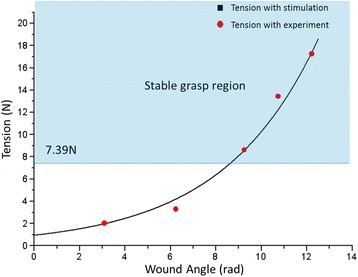
Experimental and simulated results for the tension-maintenance winder

The maximum tension at point A (T_A_) was experimentally measured at various wound angles (∅) to demonstrate convincingly that the tension-maintenance winder generates enough tension to sustain a grasp (red-dotted line in Fig. 6). The black line in Fig. 6 models the relationship between ∅ and T_A_ using the experimental data. Both the experimental and the simulated results show that the tension-maintenance winder creates enough tension for a stable grasp because the maximum tension at point A exceeds the required tension calculated in Table 1 when the wire is wound only twice around the winder [23].

### Device overview

**Fig. 7:**
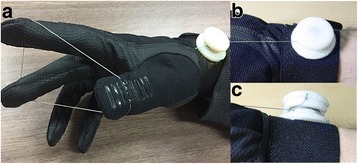
Final prototype of GRIPIT with **a** a *glove* shows tendon-routing configuration and **b**, **c** a tension-maintenance winder. Wire tension is maintained to sustain the posture by using the winder, and the tendon can be easily arranged

**Fig. 8:**
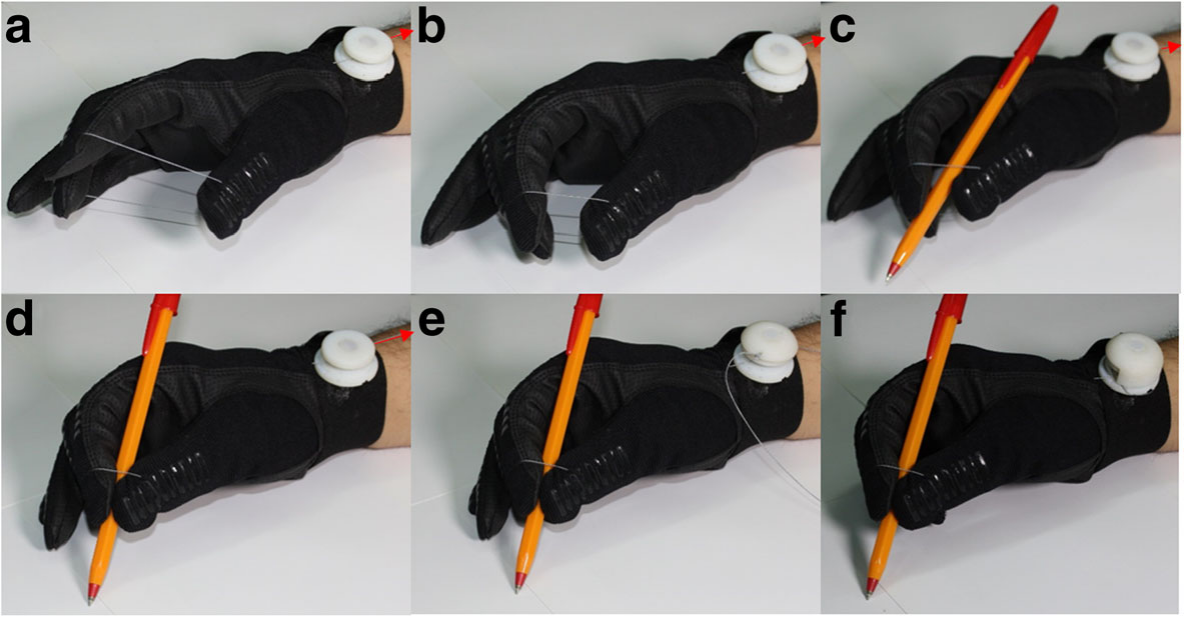
Stages of making a tripod grasp by *pulling* a single wire. **a**, **b** *Pulling* the tendon winder brings the fingers into position. **c**, **d** Further *pulling* initiates the grasp. **e**, **f** The grasp is maintained by using the spool and the winder

GRIPIT is composed of a glove with a tendon-routing mechanism and a small structure with a tension-maintenance mechanism, as shown in Fig. 7. Figure 8 shows the process of using GRIPIT. Users narrow the space between the thumb, index finger, and middle finger by pulling the wire (Fig. 8b). They put the pen into the space between the fingers using their mouth or the other hand after the space is narrowed (Fig. 8c). After the pen is well positioned in their hand, users pull the wire until sufficient tension is generated to grasp the pen (Fig. 8d). Finally, they manipulate the tension maintenance structure to sustain the grasp (Fig. 8e, f). With the above process, one wire effectively gathers the three target fingers into a single point and the generated tension is well sustained by the tension-maintenance winder.

## Results

### Experimental protocols

Table 2 describes the four people with SCI who were recruited to assess how well GRIPIT works for writing. We used the following criteria to choose the subjects: age between 20 and 40 years old, sufficient mental health to follow verbal instructions, complete loss of hand function, and C4 to C6 injuries.

**Table 2:** Summary of subject descriptions

	Subject 1	Subject 2	Subject 3	Subject 4
Gender	Male	Male	Male	Male
Age	34	33	34	31
Injury region	C5,6 ASIA-B	C5,6 ASIA-A	C5,6 ASIA-B	C5,6 ASIA-A
Year occurred	April 2005	August 2002	May 2006	May 2007
Rehabilitated duration	20 months	20 months	12 months	30 months
Accompanying damage	None	None	None	None
Complications	Bedsores	Scoliosis Orthostatic hypotension	None	Scoliosis
Time of first writing after injury	9 months later	2 years later	7 months later	3 years later

The writing evaluation was developed to compose six tasks covering both qualitative and quantitative measures (see Table 3). We assessed these performance measures in a series of experiments involving writing with GRIPIT, writing with a conventional pen holder, and writing by hand without using any mechanical aid.

**Table 3:** Summary of experimental protocols

Task	Main objective	Task type	Evaluation items
1	Qualitative measurement	Write a name	1. Appearance 2. Portability 3. Difficulty of use 4. Writing sensation 5. Fatigability 6. Legibility
2		Write a Korean letter, 청	
3		Write two given sentences	
4	Accuracy measurement	Draw circles inside of a set of boxes 50 mm and 100 mm wide	Normalized variance of the radius
5		Trace a circle, square, and triangle	Normalized root mean square value of the difference between model figures and drawn figures
6	Solidity measurement	Draw three vertical and three horizontal lines	1. Variation of the angle between hand and pen 2. Ratio between pen tip force and angle

Tasks 1 to 3 in Table 3 evaluated the qualitative performance of each writing method. The experiments consisted of writing a letter, a word, and two sentences. In Task 1, subjects were asked to write their name, because signing your name to documents is an important task of daily life. Writing the Korean character “청” was chosen as Task 2, because the character requires writing lines in several directions. For Task 3, subjects wrote the following English and Korean sentences, both of which have same meaning: “Challenges are what make life interesting; overcoming them is what makes life meaningful.” and “도전은 인생을 흥미롭게 만들며, 도전의 극복이 인생을 의미 있게 한다.” These sentences were chosen because they include more than 20 letters, which is a sufficient number to determine fatigability while writing for a long time. The subjects were asked to score on appearance, portability, difficulty of use, writing sensation, fatigability, and legibility of each writing method after performing Tasks 1 to 3. Each item was rated between a minimum score of 1 and a maximum score of 5, with a high score indicating a better result.

Tasks 4 to 6 in Table 3 evaluated the quantitative performance of each writing method in terms of the accuracy of writing (Tasks 4 to 5) and the solidity of hand posture (Task 6). Tasks 4 to 5 were designed to measure the accuracy of writing. In Task 4, subjects were asked to draw circles inside 50 and 100-mm-wide boxes. For this task, the normalized variance of the radius of all circles drawn in the given boxes (i.e., the variance of the radius divided by the mean of the radius) was measured. Although variance could be used for accuracy because it is the difference between mean and measured data, normalized variance is required in this instance because variance increases as the values of the total data increase.

However, the results of Tasks 4 depend on subjects’ spatial perception ability because the subjects must draw in an empty space without a guideline. Therefore, Task 5 was developed to measure accuracy independent of spatial perception ability. For this task subjects were asked to trace given figures, and disparities between the given figures and the drawn figures were measured. In Task 6, subjects were asked to draw three vertical lines and three horizontal lines. Pen tip force and the position of the pen were measured to calculate a ratio between the pen tip force and rotated angle of the pen during writing.

### Qualitative performance

Table 4 summarizes the qualitative writing results. The results for Tasks 1 to 3, viewed as a whole, show that GRIPIT has high advantages for writing sensation, fatigability, and legibility because of its stable grasp assistance. However, most of the subjects felt that GRIPIT was more difficult to wear and that it was more difficult to fix the pen with GRIPIT than with the conventional penholder. Subjects explained during interviews conducted after the experiments that when writing with the conventional penholder or by hand alone, the pen shakes a lot even though they apply little force during writing. These problems lead users to hold a pen vertical to the writing plane and lift their entire arm in order to reduce the torque applied to the pen. This applies high stress to the shoulder to sustain the whole arm weight. Two subjects stated that they cannot write a sentence without some type of assistive device because of high stress on their shoulder.

**Table 4:** Qualitative results for each writing method tested (C, conventional penholder; H, handwriting; G, GRIPIT)

	Subject 1			Subject 2			Subject 3			Subject 4		
Writing method	C	H	G	C	H	G	C	H	G	C	H	G
Appearance	3	3	4	3	1	4	1	5	5	4	1	5
Portability	3	-	4	3	-	4	3	-	4	4	-	4
Difficulty of pen grasp	4	-	1	4	-	2	3	-	1	4	-	1
Writing sensation	3	2	4	4	3	5	2	1	2	3	1	5
Fatigability	1	1	4	3	1	4	3	3	5	3	1	5
Legibility	2	1	3	4	1	4	2	3	4	4	3	4
Average	2.67	1.75	3.33	3.50	1.50	3.83	2.33	3.00	3.50	3.67	1.50	4.00

For all measures except difficulty of use and fatigability, higher values indicate a better result. For the difficulty of use measure, higher values indicate greater ease of use. For the fatigability measure, higher values indicate that the subject could write easily, without expending great effort. The subjects scored all of the evaluation items after the tasks

### Accuracy performance

#### Experimental data analysis method

**Fig. 9:**
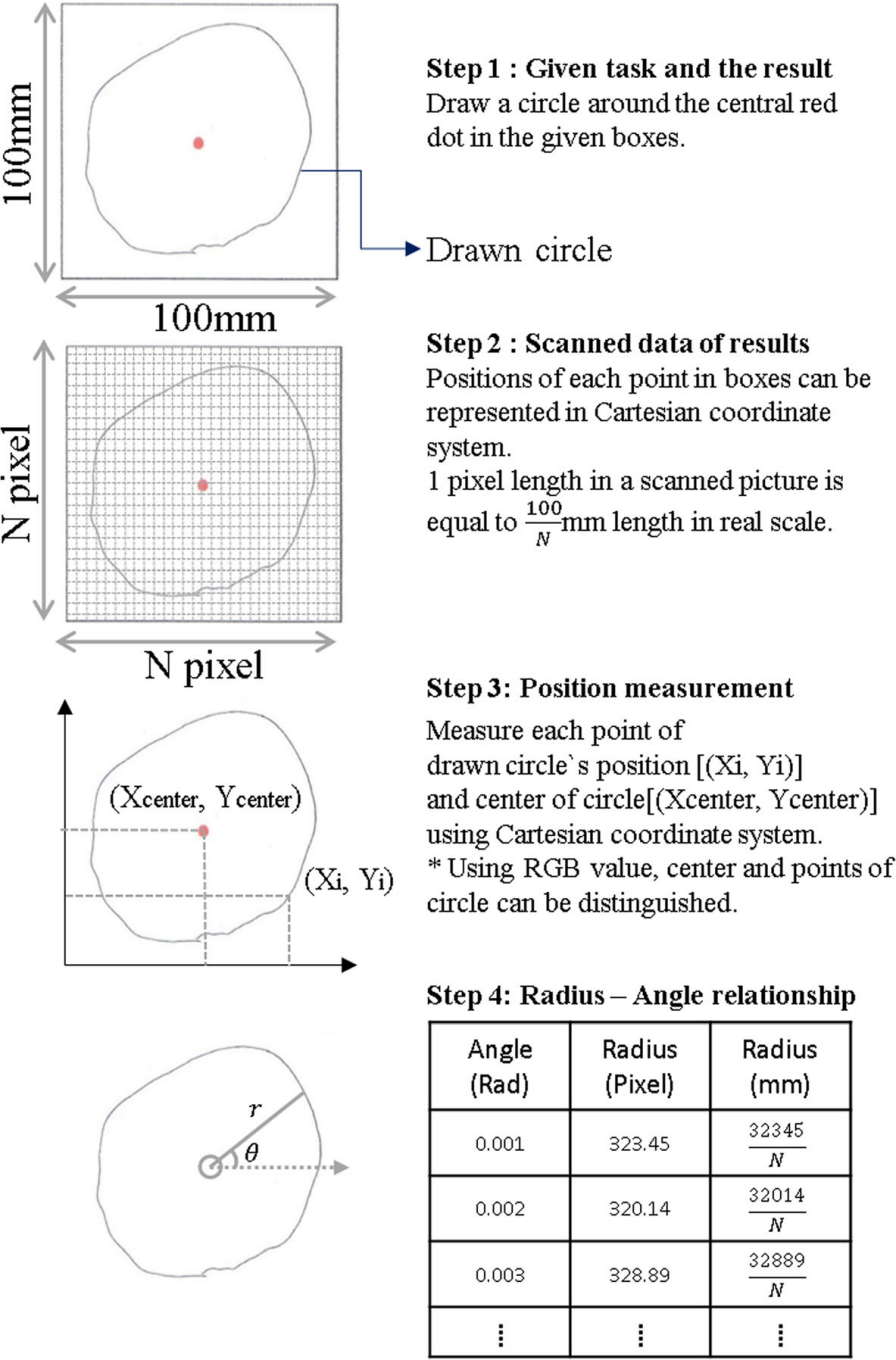
Image processing for accuracy measurement of *Task 4*

Data analysis of Tasks 4 was performed using image processing in MATLAB (Mathworks, Natick, MA, USA), as shown in Fig. 9. In step 1 of Fig. 9, subjects were asked to draw a circle around the red dot in the center of the given boxes. In step 2, the size of each box in pixel scale was measured to calculate the scale of the picture. Pixel scale is defined as the ratio between the real length and the pixel width of a box. In step 3, the box lines were removed and the positions of each point of the circle ([Xi, Yi]) and the center of the circle ([Xcenter, Ycenter]) were measured. The center of the circle and each point of the circle were distinguished by RGB value because the center is red and the drawn circle is black. In step 4, we calculated the angle and radius of each point using the positions of each point.

**Fig. 10:**
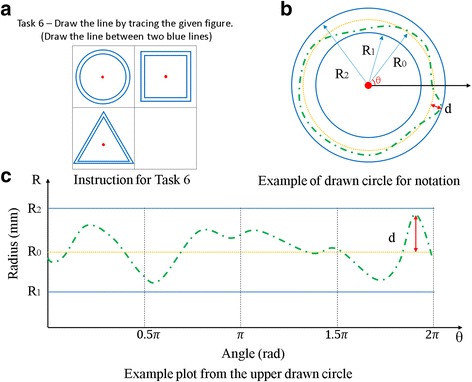
Image processing for accuracy measurement of *Task 5*. ** a** Instruction for *Task 6* **b** Example of drawn circle for notation **c** Example plot from the upper drawn circle

The image processing required for Task 5 (Fig. 10) is quite different from that required for Tasks 4 because the tasks differ so much: For Tasks 4 subjects only had to draw a circle inside a square, but Task 5 required them to trace a circle, a square, and a triangle, as shown in Fig. 10a. Figure 10b, c shows how a value that represents accuracy was obtained for the given task. The green and orange lines in Fig. 10b respectively represent a circle drawn by a subject and the baseline circle for the task. The distance between the green and orange lines ([d] in Fig. 10b, c) was measured to quantify accuracy. The normalized value of the root mean square distance was used as the accuracy indicator of Task 5 to compare these values with other tasks. The root mean square distance was divided into the size of the given figure.

### Experimental data analysis result

**Fig. 11:**
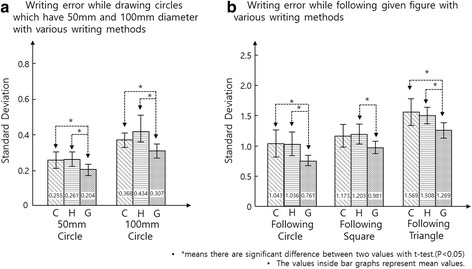
Experimental results for the accuracy tests of *Tasks 4, 5*. **a** Writing error for *Task 4*, **b** Writing error for *Tasks 5*. C, conventional penholder. G, GRIPIT. H, hand. * means there are significant differences between two values using a *t*-test. (*p* < 0.05)

Figure 11 shows that the subjects made fewer errors when drawing figures with GRIPIT than they made using a penholder, or drawing without any assistance. Data from Task 4, and 5 were analyzed statistically using a *t*-test. Writing with GRIPIT yielded a lower normalized standard deviation of radius for Task 4, and a lower normalized root mean square error between the drawn figure and the given figure for Task 5. In the case of the task, following the rectangle, the error using GRIPIT was not significantly different with use of a conventional penholder as Fig. 11 shows. The *p*-value between GRIPIT and the conventional penholder, while following the square, was 0.087, so error from GRIPIT is mostly lower than the error from the conventional penholder. Moreover, statistical analysis of the data showed that there is no significant difference between drawing with a conventional penholder and drawing without any device while performing whole tasks.

The main reason for the results obtained for Tasks 4 and 5 seems to be solidity of hand posture. The writing accuracy of non-disabled people depends on many different factors, but for people with SCI it mainly depends on postural solidity because they cannot control their fingers. GRIPIT is able to fix the pen firmly to users’ fingers via the tensioning system, whereas other writing devices cannot apply high fixation force to the pen. Task 6, which measures solidity, is discussed in detail in the next section.

### Solidity performance

#### Experimental data analysis method

**Fig. 12:**
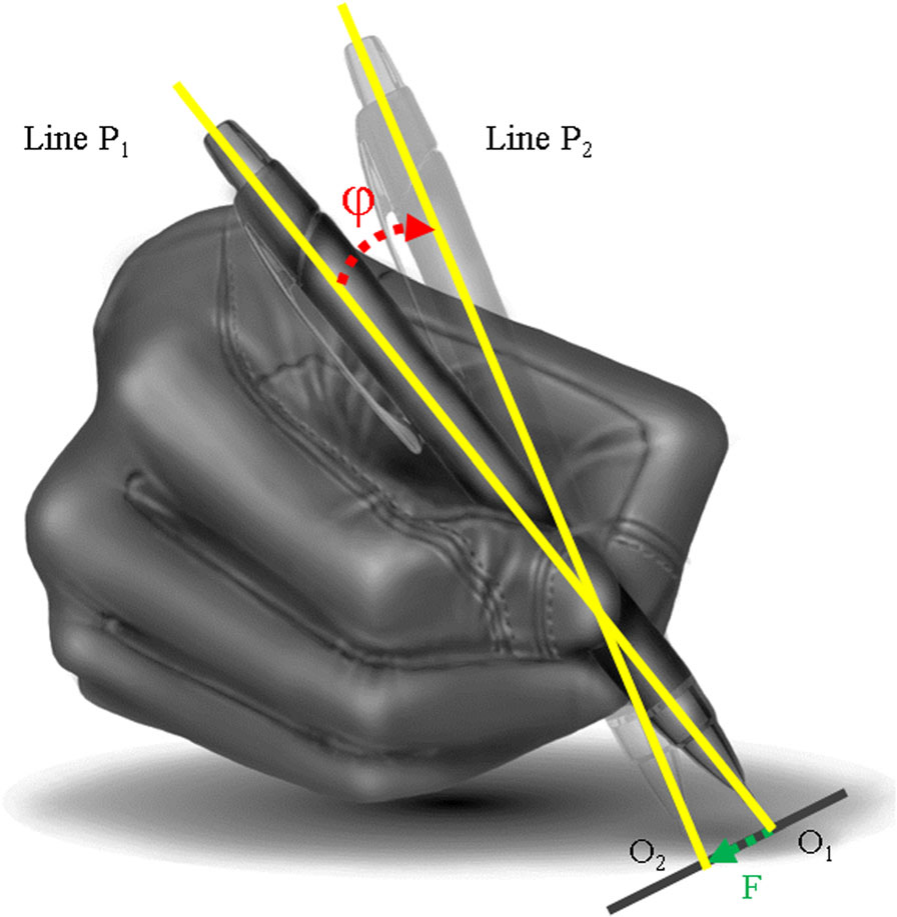
Schematic draw for solidity definition. While drawing a *line* from *O1* to *O2*, the *pen rotates* from *line P1* to *line P2*. The solidity was defined as the ration of *rotated angle of pen* (φ) and applied torque (τ)

**Fig. 13:**
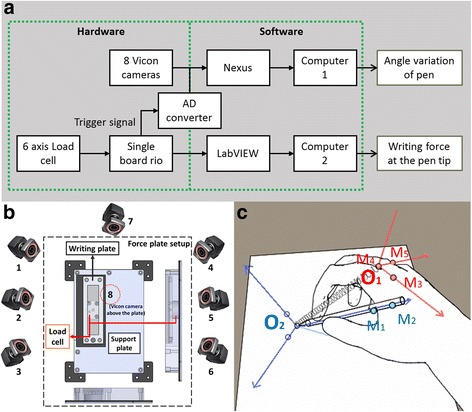
Total system construction to measure force and position of the pen *simultaneously* **a**, experimental setup to measure the *writing force* and the position of markers **b**, and marker attachment points to measure the position of pen from a *hand frame view* **c**

Solidity was measured for Task 6 by asking subjects to draw three horizontal lines and three vertical lines five times each. The ratio of applied force (F in Fig. 12) and the rotated angle of the pen (φ in Fig. 12) was used as an indicator of solidity [29, 30]. The system for simultaneously measuring force and position data is shown in Fig. 13a.

We used a load cell to measure the force and motion capture system to measure the variation of the angle. The force measurement stage shown in Fig. 13b was constructed using a Nano17 (ATI, Apex, NC, USA), a writing plate, and a support plate. The six-axis load cell was attached under the writing plate to measure the writing force, which was defined as the friction force between the pen and the writing plate. The support plate, on which subjects could lean their arm, was attached parallel to the writing plate to exclude the arm weight from the writing force. Data were collected using a Single Board Rio and LabVIEW (National Instruments Corporation, Austin, TX, USA).

The Vicon system (Vicon, LA, USA), which detects the position of reflective markers with infrared light, was used to obtain information about the location of the pen and subjects’ hand. Two markers (M_1_, M_2_, Fig. 13c) were attached to the pen to track its position, and three markers (M_3_–M_5_, Fig. 13c) were attached to the hand to create an x-axis, a y-axis, and an origin for the moving system frame. Eight Vicon cameras were used to measure the markers while performing the experiment. Each of the three cameras were placed on the left and right sides of the desk, one camera was placed on the top, and one on the front of the desk to continuously check the overall markers.

We used an analog output function from a Single Board Rio device for data synchronization. After the experiment, we collected the position data from nexus and force data from LabVIEW. Using the trigger signal in the force data, we synchronized the data and calculated the ratio between the angle variation and writing force.

#### Experimental data analysis result

**Fig. 14:**
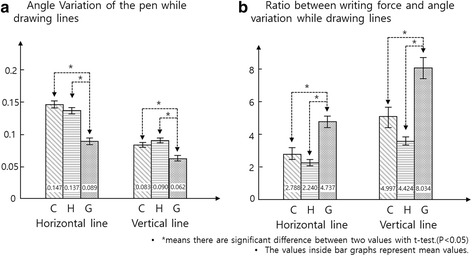
Experimental results for Task 6. **a** Contact angle variation while drawing with a conventional penholder (C), hand drawing (H), and drawing with GRIPIT (G); small contact angle variation means pen doesn’t shake a lot while writing. **b** Ratio between pen tip force and contact angle during drawing with a conventional penholder (C), hand drawing (H), and drawing with GRIPIT (G); Large force/contact angle ratio means pen doesn’t shake a lot even high force is applied on the pen. . * means there are significant differences between two values using a *t*-test. (*p* < 0.05)

The ratio of angle variation and writing force, which is the variable for solidity, was calculated using MATLAB, and the results are plotted in Fig. 14. Angle variation and force/angle ratio results for all subjects show that the writing performance of GRIPIT is significantly better than the performance obtained with other writing methods, when statistical analysis was performed. Moreover, statistical analysis of the data showed that there is no significant difference between drawing with a conventional penholder and drawing without any device while performing whole tasks. A large value for the force/angle ratio means that users can write without their hands shaking, even when they apply high force to the pen.

## Discussion

We developed GRIPIT to assist SCI people to make tripod grasp for them to go back to occupation or school because this grasp is suitable to make stable grasp force and moment balance while grasping small objects or various pen. The device is designed to be compact, lightweight, and simple by using the underactuated tendon-driven mechanism and the tension-maintenance winder. After the device development, we devised new assessments to measure writing ability from various viewpoints including both qualitative and quantitative methods, while most conventional assessments include only qualitative methods or simple time measuring assessments. Qualitative assessment results show that GRIPIT is more complicated to wear and use than conventional writing assist devices. Experimental participants reported that process of using GRIPIT, consisting of wearing a glove, fixing a glove, inserting a pen, pulling a wire, and manipulating the tension maintaining part, is more complicated than the process of a conventional penholder that consists of fixing a pen into a hole and attaching the splint to the body. However, responders claimed writing with GRIPIT is preferred because it improves the sensation of writing, fatigability, and legibility because it applies a high fixation force to the pen and compensates torque generated by contact forces. Quantitative measurements of writing accuracy and solidity of hand posture show that GRIPIT yields better results than other methods. Writing with a conventional penholder and handwriting show similar results, which means that a conventional penholder is not that useful compared to the handwriting. However, GRIPIT assists users to write accurately without shake of the pen even high force is applied on the pen as the evaluation results show. We believe that reducing the shake of the pen while writing is the most important factor for people with SCI because they cannot use their fingers to control the pen and have to use their arm due to their paralyzed fingers. This factor also attributes writing with GRIPIT to get better results in the total evaluations because GRIPIT assists users to apply sufficient grasp force and form tripod grasp to get high resistance in the torque applied by pentip force.

This study has two major limitations in terms of device development and writing assessment. Although we developed device to assist grasp using a single wire for convenience, tension-maintenance winder requires several kinds of process to prepare grasp and makes the using process complicate according to the interview from the qualitative assessment. Although subjects can wear and use GRIPIT alone, they insisted that it would be more useful if the process of using GRIPIT was simplified. The device will be improved using motor or other tension-maintenance mechanism for the future works. Another limitation of this study, which is concerned with assessment, is a limited sample size; nevertheless, this study should suffice to notify GRIPIT assist users to write better than using other devices and the assessment method is useful for writing evaluation. More experiments with improved GRIPIT using motor or other tension-maintenance mechanism should be considered in future studies.

## Conclusions

We developed a glove-type assist device named GRIPIT to assist people with SCI to better use their hands. GRIPIT helps people to grasp tools by using manual actuation to apply force to the fingers via a single wire. In contrast, conventional assist devices only fix tools to the hand by fitting them into a hole on the device and then attaching the device to the hand with Velcro. GRIPIT also has a simpler design and an easier actuation method than other exoskeleton robots, because it consists of only a single glove and a single wire.

GRIPIT has a high potential to increase quality of life for people with hand paralysis because it can help users to grasp a range of tools, whereas conventional devices can accept only the products that fit into their hole. Moreover, GRIPIT can be easily extended to form other grasp postures by changing the routing points of its wire. GRIPIT can be extended to assist most of the hand functions required in daily life by designing new forms of its glove for various grasp postures and by improving its wearability and ease of use.


Additional file 1: GRIPIT wearing process video of spinal cord injured person. (WMV 43662 kb)


## Abbreviations

C: Conventional penholder
DOF: Degrees of freedom
G: GRIPIT
H: Hand writing
SCI: Spinal cord injury
